# Core-Shell Structured Electro- and Magneto-Responsive Materials: Fabrication and Characteristics

**DOI:** 10.3390/ma7117460

**Published:** 2014-11-21

**Authors:** Hyoung Jin Choi, Wen Ling Zhang, Sehyun Kim, Yongsok Seo

**Affiliations:** 1 Department of Polymer Science and Engineering, Inha University, Incheon 402-751, Korea; E-Mail: wlzhang@inha.ac.kr; 2 Polymer Processing Technology Team, LG Chemical Ltd./Tech Center, Daejon 305-738, Korea; E-Mail: shkim@lgchem.com; 3 Research Institute for Advanced Materials, Department of Materials Science and Engineering, Seoul National University, Seoul 151-742, Korea

**Keywords:** core-shell structured materials, field-responsive materials, ER/MR application

## Abstract

Core-shell structured electrorheological (ER) and magnetorheological (MR) particles have attracted increasing interest owing to their outstanding field-responsive properties, including morphology, chemical and dispersion stability, and rheological characteristics of shear stress and yield stress. This study covers recent progress in the preparation of core-shell structured materials as well as their critical characteristics and advantages. Broad emphasises from the synthetic strategy of various core-shell particles to their feature behaviours in the magnetic and electric fields have been elaborated.

## 1. Introduction

Core-shell structured materials belong to an interesting category of designed targeted particles with multi-functions and a variety of expected structures [1,2,3]. Core-shell type particles have outstanding advantages over pure particles and potentially enable potential applications in nano-biotechnology, drug delivery, sensors and stimulus-responsive systems [4,5,6,7,8]. This study focuses on recent advances in the development of mainly inorganic core-shell structured particles for potential use in electrorheological (ER) and magnetorheological (MR) applications that require a material response under an applied electric or magnetic field, respectively.

“Smart” functional materials are increasingly used in a wide range of applications because of their controllable reaction to external environmental stimuli, such as light, temperature, pH, stress, electric or magnetic field,* etc.* [9]. Electro- and magneto-responsive particles belong to a class of smart suspensions that can undergo a reversible phase transition between a liquid-like and a solid-like state in the presence of an electric or magnetic field. The field-stimulated structure modulates the rheological properties, which are called ER or MR effects [10,11]. An ER fluid is typically composed of polarizable particles with a large dielectric constant dispersed throughout an insulating carrier liquid. An MR fluid is generally a suspension of magnetizable particles in a non-magnetic medium. The intelligent ER/MR materials have been utilized in a wide range of engineering applications, including microfluidic devices, vibration damper systems, engine mounts, clutches, human muscle stimulators, advanced polishing technology, and drug delivery [12,13,14,15]. Diverse materials with high dielectric constants, good dispersion stabilities and low densities, such as dielectric inorganics, semi-conducing polar polymers, novel carbonaceous materials, and their composites have been investigated as ER particles. Recently, graphene/graphene oxide (GO)-based materials have been examined as novel ER candidates because of their good mechanical strength and special layer structures [16,17,18,19]. Graphene, a two-dimensional layer of carbon atoms, possesses excellent physical and chemical properties that could enable applications in biosensors, electrochemical energy storage, solar cells, and field emission devices [20]. GO has attracted significant attention in recent years as a promising precursor of graphene, due to its hydrophobic basal plane and a number of hydrophilic groups on it, which convey amphiphilic properties and permit facile chemical modification [16,17,18,19].

The ER activity depends on the morphological factors associated with preparing and maintaining a good dispersion of the materials. In this respect, particles with a core-shell structure have shown promise in ER applications because of their remarkable ER properties, low cost and well controlled particle size [21,22]. Diverse core-shell structured particles prepared through different mechanisms have been reported to show enhanced ER effects. A class of interesting particles composed of polystyrene (PS) microspheres coated with GO sheets was prepared using π–π stacking interactions [23]. Liu* et al.* [24] used core-shell structured polyaniline (PANI)-coated anisotropic snowman-like poly(methyl methacrylate) (PMMA) particles prepared with an anionic surfactant as ER fluid suspension particles. Yin* et al.* [25] have reported MR properties of core-shell structured GO-wrapped Titania dielectric microspheres prepared through an electrostatic absorption method.

Several MR fluids have been used in engineering applications. Carbonyl iron (CI, almost pure iron) particles are the most common MR materials due to their excellent magnetic properties and appropriate particle sizes; however, the high density of CI particles, which reduces the stability of dispersion against sedimentation, has limited their extensive technological applications. Several approaches to improving the dispersion stability has been tested, including the addition of dispersion stabilizers or additives, the use of a viscoplastic carrier medium, and the modification of CI particles with either polymers or inorganic coatings [26,27,28,29,30,31]. Coating CI microbeads with an inorganic material can increase the durability of the particles under acidic conditions, leading to a long lifetime. Liu* et al.* [32,33] have reported core-shell structured silica-encapsulated CI particles with a low particle density, enhanced oxidation-resistance and anti-acidic properties. Carbon nanotubes (CNTs) and graphene oxide (GO) display similar self-assembly capabilities [34,35], thereby motivating the introduction of a surface layer composed of multi-walled CNTs (MWCNTs) or GO onto the CI particles, stabilized by 4-aminobenzoic acid (PABA) as a grafting agent [31,36]. A dense layer of MWCNTs has been coated onto the CI surfaces using dual ultra-sonication and a solvent casting method (The solvent-casting method is comprised of two steps: we prepared a MWCNT thin layer first coated under ultrasonication on the surface of PABA (4-aminobenzoic acid) pre-treated CI particles. Obtained particles (S-MWCNT-CI) were then added to a prepared homogenous COOH-MWCNT (C-MWCNT)/distilled-water dispersion via mild ultrasonication and underwent vigorous stirring, then poured into a large beaker containing a silicone oil bath at a temperature of 95 °C and underwent intense stirring to form a water-in-oil emulsion. Such a high temperature, along with a high stirring velocity was able to evaporate the water completely, thus accomplishing the process of loading dispersed MWCNT on the surface of S-MWCNT-CI particles.). The GO- or MWCNT- coated CI particles were found to display magnetic properties similar to those of pure CI particles but with improved dispersion stabilities due to reduced particle density [37]. In addition, MWCNTs could be coated onto polymeric-coated CI particles in a sequential coating process [29,37]. Compared to the smooth surface of the polymer-coated CI particles, the MWCNT–polymer-coated CI particles featured rough surfaces, which improved the suspension stability. Iron oxides (such as Fe_2_O_3_ and Fe_3_O_4_) have been used as MR materials, because of their much lower density than CI particles [38] and their relatively good magnetic properties. Kim* et al.* [39] prepared core-shell-structured Fe_2_O_3_-coated polystyrene particles using an eco-friendly Pickering emulsion polymerization method; and they examined the MR properties of the particles dispersed in silicone oil under various magnetic field strengths. This study reviews progress in the core-shell structured materials for use in ER/MR applications.

## 2. Fabrication and Field Response

### 2.1. Electrostatic Attraction

Electrostatic attraction has been employed as a rapid method for fabricating a variety of composites. As mentioned, bare GO sheets with polar groups and a high surface area have been considered as interesting ER candidates. The intrinsic properties of pure graphene sheets, including high electrical conductivity and particle stacking aggregation during the robust shearing process, may cause electric shorts even at low particle concentration, thereby limiting their ER effects. Therefore, GO-based composites with a high dielectric constant and a relatively low electric conductivity are desirable for use in ER applications.

**Figure 1:**
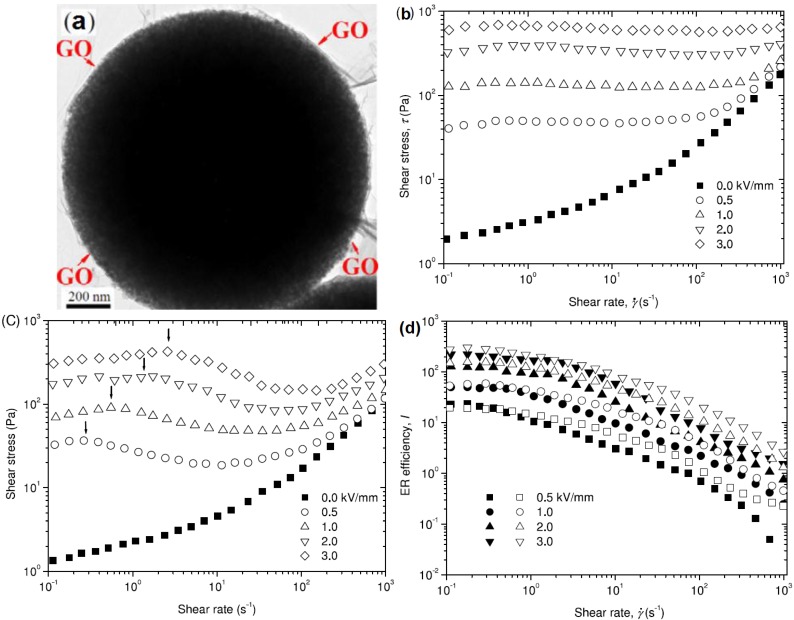
(**a**) TEM image of GO-wrapped TiO_2_ microspheres. Also shown are the flow curves obtained from ER fluids based on (**b**) GO-wrapped TiO_2_ and (**c**) bare TiO_2_ microspheres (the arrows indicate the maximum stress points); (**d**) the ER efficiencies of ER fluids based on the GO-wrapped TiO_2_ (open symbols) and pure TiO_2_ microspheres (solid symbols) [25] (Reproduced with permission from *Nanotechnology*, published by IOP, 2014).

Yin* et al.* [25] reported the preparation of core-shell structured GO-wrapped titania dielectric microspheres using a facile electrostatic attraction method for fabricating an ER suspension. As shown in Figure 1a, the surfaces of the positively charged titania microspheres were covered with the soft, negatively charged GO layers. The shear stress within the GO-wrapped titania microspheres’ suspensions displayed a broad stable plateau over a wide shear rate range, unlike the bare titania microsphere suspension, as shown in Figure 1b,c. The ER efficiency, *I*, defined as *I* = (τ_E_ − τ_0_)/τ_0_, where τ_E_ is the shear stress under an applied electric field; and τ_0_ is the shear stress without an electric field, is a critical parameter for evaluating the change in ER behavior in the presence or absence of an electric field stimulus. The GO-wrapped titania microspheres-based ER fluid displayed a higher ER efficiency compare with the bare titania microspheres-based ER fluid, confirming the positive ER effects of the GO sheets.

Green* et al.* [40] recently showed that a suspension of micrometer-sized polystyrene (PS) particles in a PDMS liquid, mixed with small (1 wt%) amounts of a nanocage, sulfonated polyhedral oligomeric silsesquioxane (s-POSS), exhibited significant electrorheological (ER) behavior. This behavior was associated with the formation of a thin adsorbed layer of s-POSS onto the surfaces of PS and the subsequent formation of polarization-induced aggregates, or structures, responsible for the ER effect in an applied electric, E, field. The interfacial adsorption of a layer of dipolar molecules onto the surface of PS particles in a PDMS medium changed a previously ER inert suspension (PS/PDMS) to a viable ER fluid: s-POSS/PS/PDMS. In the subsequent study, they showed that by adding small concentrations (less than 2 wt%) of s-POSS to the ER fluid, sulfonated-PS (s-PS)/PDMS, resulted in the formation of a superior ER suspension, s-POSS/s-PS/PDMS, exhibiting a significant improvement in ER activity of over 200% at moderate fields [41]. This behavior was not readily rationalized in terms of current ER theories, which suggests that the properties of the shell, dielectric and conductive, largely determine the yield stress of the system. This result indicates that the conductive properties of the core are very important, otherwise the ER behavior of the s-POSS/PS/PDMS and the s-POSS/s-PS/PDMS suspensions would be comparable.

### 2.2. Self-Assembly Method

**Figure 2:**
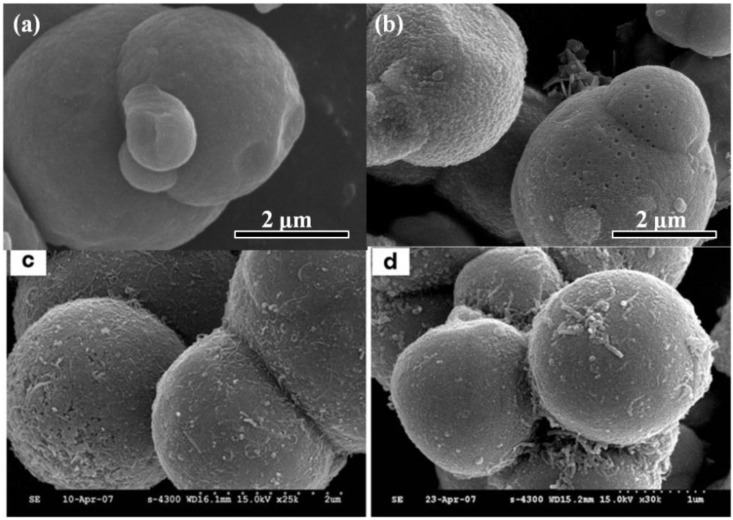
SEM images of (**a**) bare CI particle [31]; (**b**) GO/CI particle [31]; (**c**) MWCNT-coated CI particles prepared with sonication for 12 h [36]; or (**d**) for 24 h [36]. (Reproduced with permission from *J. Appl. Phys.*, published by AIP, 2014 and from *Colloid Polymer Science*, published by Springer, 2010).

Self-assembly approaches are used to prepare a variety of materials [42]. The self-assembling properties of MWCNTs and GO sheets, may be adjusted by modifying the surfaces of CI particles using 4-aminobenzoic acid (PABA) as a grafting agent in the MR dispersing phase to improve the stability of the CI-based MR systems. The surface of pristine CI particles tends to be smooth and the size distribution tends to be polydisperse, as shown in Figure 2a. After modification with PABA, the surfaces of the CI particles presented amino groups, that could react with the -COOH or other functional groups present on the GO sheets or modified MWCNTs. The GO sheets or MWCNTs could then coat the surfaces of the CI particles under sonication [31,36]. The GO/CI and MWCNT-wrapped CI particles displayed rough surfaces, as shown in Figure 2b,c. The use of sonication can avoid GO sheet and MWCNT aggregations; however, Fang* et al.* [36] demonstrated that longer sonication times did not improve the quality of the dense MWCNT coating on the CI particle surfaces, as shown in Figure 2d.

### 2.3. Solvent Casting Method

**Figure 3:**
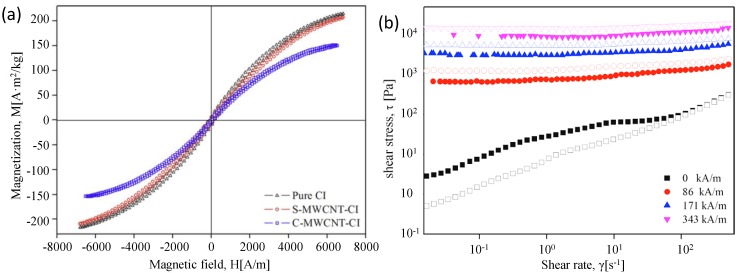
(**a**) VSM (Vibrating Sample Magnetometry) curves obtained from the pure CI, S-MWCNT-CI, and C-MWCNT-CI particles; (**b**) shear stress curves obtained from the C-MWCNT-CI (closed) and pure CI (open) particles-based MR suspensions [37]. (Reproduced with permission from *Colloids & Surfaces A: Physicochemical and Engineering Aspects*, published by Elsevier, 2012).

As mentioned, the long sonication time is not favorable for achieving a denser coating of the MWCNT layer. Solvent casting methods have been applied as the second coating step (C-MWCNT-CIs) to CI particles pre-treated with PABA and subsequently treated with MWCNTs (S-MWCNT-CI) prior to the application of the second solvent casting coating, yielding C-MWCNT-CIs [37]. The magnetization curves of the samples, shown in Figure 3a, reveal the magnetic properties of these particles. Small differences between the pure CI particles and the S-MWCNT-CI particles were observed; however, after the introduction of MWCNTs via the solvent casting method, the value of magnetization saturation drops off sharply, possibly due to the oxidation of the CI particles during the solvent casting process. The presence of GO sheets or MWCNTs is expected to reduce the density of CI particles and improve their dispersion stability. Figure 3b shows that the C-MWCNT-CI suspension exhibited a higher shear stress than the pure CI suspension under a free magnetic field, as expected based on the particle surface roughness. Under an applied external magnetic field, MR particles become polarized and contact one another, leading to an increase in the shear stress. The shear stress of the C-MWCNT-CI suspension was slightly lower than that of the pure CI particles-based MR suspension, in agreement with the relatively low magnetization saturation value. MWCNTs nest on the surface of CI particles. In the case of MR characterization, both yield behavior and elastic property decreased due to the reduced magnetic property. Improvement in sedimentation was observed due to the reduced density along with the rough surface. Compared with the complexity of coating polymeric shell, coating MWCNTs proved to be easier [37].

### 2.4. Sol-Gel Method

**Figure 4:**
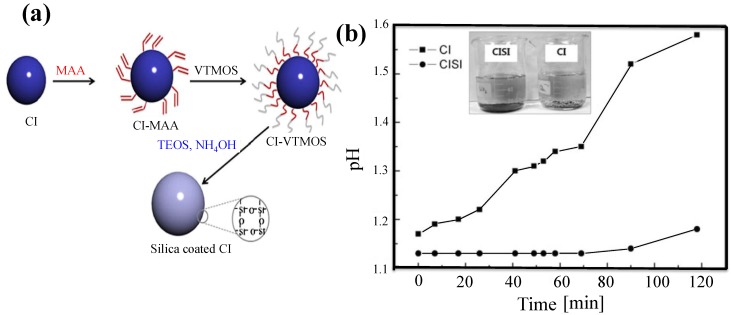
(**a**) Schematic diagram illustrating the synthesis of the CISI particles [33]; (**b**) Resistance of the CISI and pure CI particles to acid degradation [33]. (Reproduced with permission from *Colloids and Polymer Science*, published by Springer, 2011).

Silica is an important inorganic material due to its stable chemical and physical properties, ease of fabrication, and low price. Liu* et al.* [32] reported the generation of core-shell structured silica-encapsulated (silane grafted) CI particles (CISI) via an attractive sol-gel method, as shown in Figure 4a. The presence of silica protected the CI particles from oxidation in an HCl solution. The pH of the CISI particles incubated in this acidic solution remained stable, confirming the effective anti-acid properties of the inorganic silica coating, as shown in Figure 4b. Although the magnetic properties of the CI-SiO_2_ particles were lower than those of the pure CI particles, their MR fluid performed well with solid-like properties and good dependence of storage and loss modulus on the intensity of magnetic field [33].

### 2.5. Eco-Friendly Pickering Emulsion Polymerization

**Figure 5:**
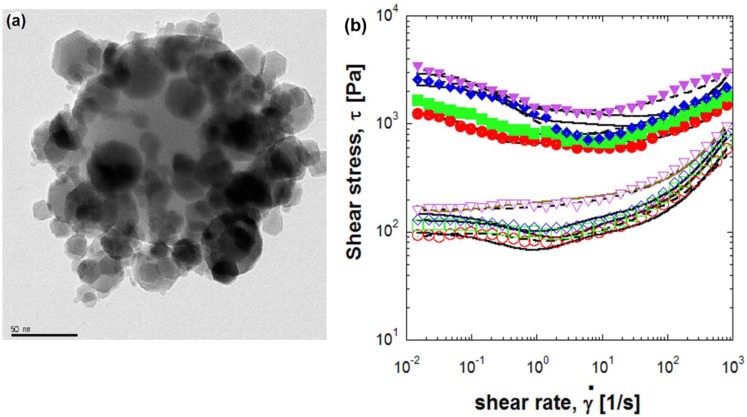
(**a**) TEM image of the PS/Fe_2_O_3_ particles; (**b**) shear stress* vs.* shear rate of PS/Fe_2_O_3_ (open symbols)- and bare Fe_2_O_3_ (closed symbols)-based MR fluids. The dotted and solid lines indicate fits obtained using the CCJ model and the Seo-Seo model, respectively [39]. (Reproduced with permission from *Langmuir*, published by ACS, 2013).

Iron oxide nanoparticles have been widely studied for use in information storage, lithium battery, and water treatment applications [43,44,45]. Magnetic particles (such as Fe, Fe_2_O_3_ and Fe_3_O_4_) systems have been investigated as MR materials [46,47]. Compared with the CI particles, iron oxide particles have a much lower density but significant magnetic properties. Kim* et al.* [39] fabricated core-shell structured magnetic polystyrene (PS)/inorganic particles using an eco-friendly Pickering emulsion polymerization method applied to nanosized Fe_2_O_3_ particles as a solid stabilizer. Here, the solid inorganic particles were chosen as the surfactant in place of conventional organic surfactants. As shown in Figure 5a, the PS nanospheres were surrounded by Fe_2_O_3_ nanoparticles.

The MR properties of the pure Fe_2_O_3_ and PS/Fe_2_O_3_ particles were investigated and compared, as shown in Figure 5b. The saturation magnetization of the pure Fe_2_O_3_ was higher than that of the PS/Fe_2_O_3_ composites due to the introduction of non-magnetic PS spheres. As expected, the PS/Fe_2_O_3_-based MR fluid displayed a lower shear stress compared with that of the bare Fe_2_O_3_-based MR fluid under the same magnetic field strength and over the full range of shear rates. The shear stress curves obtained from the Fe_2_O_3_-based MR fluid decreased at lower shear rates, unlike the corresponding curves obtained from the MR systems based PS/Fe_2_O_3_ and commercial CI particles.

The peculiar behavior represented by the flow curves could not be fitted using the Bingham fluid model, which is frequently used to predict the yield stress (Figure 5b). Another model, the Cho-Choi-Jhon (CCJ) model, was used to describe the structural variation as a function of the shear flow:

(1) $$\tau =\;\frac{\tau_{\text{dy}}}{\left(1+{\left(t_{1}\dot{\gamma }\right)}^{\alpha }\right)}+\eta_{\infty }\left(1+\frac{1}{{\left(t_{2}\dot{\gamma }\right)}^{\text{β}}}\right)\dot{\gamma }$$

where t_1_ and t_2_ are time constants; $\dot{\gamma }$ is the shear rate; τ_dy_ is the yield stress; and η_∞_ is the viscosity at high shear rates [48]. The first term on the right-hand side represents the shear stress behavior at low shear rates and indicates that the shear stress decreases with the shear rate after exhibiting yield behavior. The second term describes the shear thinning behavior at high shear rates. This model could describe the deformation behavior of the fluids; however, the yield stress obtained from this model fit was the dynamic yield stress. A detailed description of this CCJ model using six parameters can be found in previous publications [46,48]. As shown in Figure 5b, the solid lines obtained from the CCJ model agreed well with the experimental shear stress curves. The yield stress of the ER fluids, however, was equal to the shear stress required to initiate a shear flow from the resting fluid state, that is, the yield stress for an undisrupted fluid. Fossum *et al.* [49] noted that the yield stress for an ER fluid under continuous shear and under the application of a large electric field should be the static yield stress, τ_sy_, which can differ significantly from the dynamic yield stress. There are two yield stresses for MR fluids: a dynamic yield stress, τ_dy_, corresponds to the stress of the MR fluid that is completely broken down under continuous shearing, and the static (or frictional) yield stress, τ_sy_, is the minimum stress required to cause the suspension to flow. To predict the static yield stress, τ_sy_, rather than the dynamic yield stress, τ_dy_, Seo and Seo proposed another model:

(2) $$\tau =\tau_{\text{sy}}\left(1-\frac{\left(1-\exp\left(-a\dot{\gamma }\right)\right)}{\left(1+(a{\dot{\gamma )}}^{\alpha }\right)}\right)+\eta_{\infty }\dot{\gamma }$$

where τsy is the static yield stress, η∞ is the shear viscosity, *a* is the reciprocal shear rate (*a* = ${\dot{\gamma }}^{-1}$ for mesostructure deformation) above which the fluid flows with a plastic viscosity η_∞_, and α is an empirical parameter, a power-law index used to determine the degree of shear thinning [50]. As shown in Figure 5, if the shear stress overcomes the magnetic field forces that hold the aggregates together, fibrillated chain structures of particles will be destroyed and the MR fluid will flow. Hence, the shear stress decreased with the shear rate, but the broken structures tended to reform an aligned mesostructure (fibril chains or columns) under an applied magnetic field. The shear stress decreased after reformation because the reformed structures were not as complete as those that formed prior to the application of shear flow [51,52,53]. Though the fits obtained from the Seo-Seo model agreed with the CCJ model, as shown in Figure 5b, the static yield stress obtained from the Seo-Seo model differed significantly, especially for the core-shell structured Fe_2_O_3_/PS particle suspension fluids. Additional details are under investigation and will be described in a forthcoming publication.

## 3. Conclusions

Core-shell structured particles for use in ER or MR fluids were successfully prepared using several methods, including electrostatic attraction, self-assembly, solvent casting, sol-gel and the Pickering emulsion method. The well-designed particles formed a suspension with a lower density, enhanced dielectric constant, and/or improved dispersion stability. Not only the coating materials’ properties but also the core materials’ properties, especially the electrical conductivity, can affect the polarization of the core-shell particles to result in enhanced ER/MR properties. Further studies to enhance ER/MR properties and improve tstability are underway and will be reported in the future.
